# A rare pulmonary hamartoma: fibroleiomyomatous hamartoma

**DOI:** 10.1186/s40792-016-0184-z

**Published:** 2016-06-02

**Authors:** Yoshinobu Ichiki, Junji Kawasaki, Takayuki Hamatsu, Taketoshi Suehiro, Ryo Shibuya, Atsuji Matsuyama, Fumihiro Tanaka, Masanori Hisaoka, Keizo Sugimachi

**Affiliations:** Department of Chest Surgery, Onga Nakama Medical Association Onga Hospital, 1725-2 Ooaza-Ozaki Ongacho, Onga-gun, Fukuoka, 811-4342 Japan; Department of Surgery, Onga Nakama Medical Association Onga Hospital, Onga-gun, Fukuoka, Japan; Department of Emergency, Onga Nakama Medical Association Onga Hospital, Onga-gun, Fukuoka, Japan; Department of Pathology and Oncology, School of Medicine, University of Occupational and Environmental Health, Kitakyushu, Japan; Second Department of Surgery, School of Medicine, University of Occupational and Environmental Health, Kitakyushu, Japan

**Keywords:** Lung tumor, Hamartoma, Surgery, Thoracoscopy, VATS

## Abstract

Pulmonary hamartomas are more common than expected because they are usually asymptomatic and are either discovered on routine chest radiography or when they are noted incidentally in approximately 0.25 % of autopsies. In contrast, pulmonary fibroleiomyomatous hamartoma, which consists of interlacing bundles of smooth muscle cells admixed with fibrous tissue and numerous tubular or cleft-like epithelial inclusions, is a rare type of hamartoma. Controversy exists regarding the pathogenesis of this tumor. We herein present a rare case of a 68-year-old male patient without a pre-existing smooth muscle tumor, who underwent resection for a tumor that was considered to be a true pulmonary fibroleiomyomatous hamartoma.

## Background

Hamartomas are a relatively common form of benign lung tumor. Fibroleiomyomatous hamartomas, on the other hand, are rare. Pulmonary fibroleiomyomatous hamartoma was first described as “diffuse fibroleiomyomatous hamartomatosis” by Cruickshank et al. in 1953 [1]. To date, only five cases of solitary pulmonary fibroleiomyomatous hamartoma have been reported [2, 3]. These tumors represent an important group of benign pulmonary tumors which classically present as incidental “coin lesions” and which must be differentiated from primary and metastatic malignant neoplasms. We herein present a rare case of a patient with a solitary pulmonary fibroleiomyomatous hamartoma.

## Case presentation

**Fig. 1:**
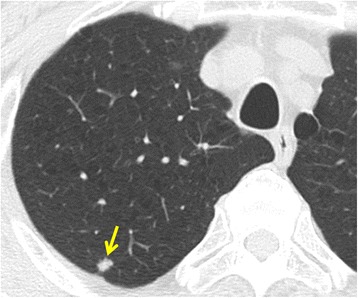
Chest computed tomography (CT) revealed a well-demarcated solid nodule in the right upper lobe adjacent to the pleura, which measured 1.0 cm in diameter

The patient was a 68-year-old asymptomatic male who visited our hospital after an abnormal shadow was suspected on a chest X-ray. He had undergone surgery for colon cancer 8 years previously. He had no history of smooth muscle tumors nor did he have any history of occupational exposure to silica, beryllium, or asbestos. Chest computed tomography (CT) revealed the presence of a well-demarcated solid nodule in the right upper lobe adjacent to the pleura, which measured 1.0 cm in diameter (Fig. 1). A systemic CT scanning examination revealed no tumors other than this pulmonary tumor.

**Fig. 2:**
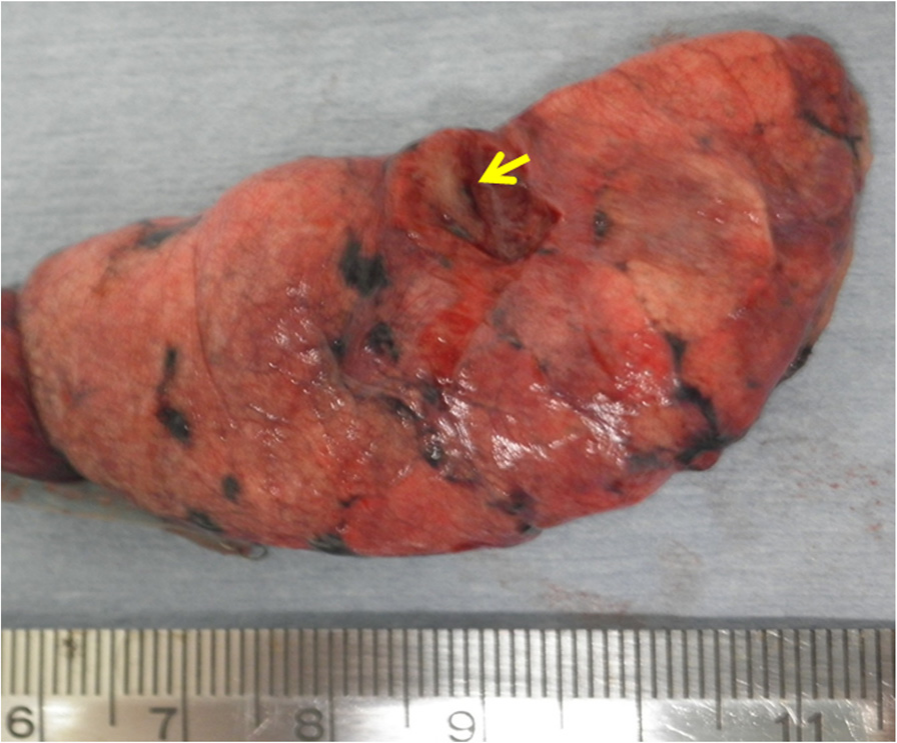
The nodule was firm, solid, yellow-white in color on sectioning, and was not associated with the bronchus or macroscopically visible bronchioles

**Fig. 3:**
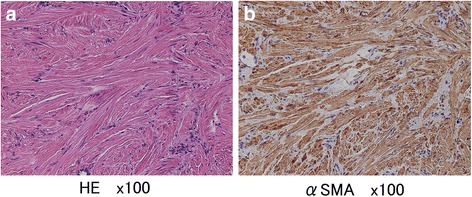
**a** The histopathological findings revealed the proliferation of spindle cells with eosinophilic cytoplasm arranged in an interlacing bundle and bronchial epithelium extending into the alveolar septa in the pulmonary parenchyma without a cartilaginous or adipose element. Mitotic figures were not observed. **b** An immunohistochemical analysis revealed that the spindle cells were positive for smooth muscle cell markers such as alpha-smooth muscle actin (SMA)

We performed a wedge resection of the right upper lobe by video-assisted thoracoscopic surgery (VATS). Three ports were placed in the left lateral decubitus position. The lung was deflated to confirm the right lung nodule. A linear stapling device was used to resect the lung nodule. The nodule was firm, solid, yellow-white in color on sectioning (Fig. 2), and was not associated with the bronchus or macroscopically visible bronchioles. A frozen section revealed the proliferation of fibromuscular tissue without mitotic figures, suggesting that it was a benign tumor, such as a leiomyoma. Consequently, lobectomy was not performed. The postoperative course was uncomplicated. The histopathological findings revealed the proliferation of spindle cells with eosinophilic cytoplasm arranged in an interlacing bundle and bronchial epithelium extending into the alveolar septa in the pulmonary parenchyma without a cartilaginous or adipose element. Mitotic figures were not observed (Fig. 3a). An immunohistochemical analysis revealed that the spindle cells were positive for alpha-smooth muscle actin (SMA) (Fig. 3b) and HHF-35, focally positive for desmin and negative for estrogen receptor, progesterone receptor, Melan A, and HMB-45. No Epstein-Barr virus-infected cells were identified by in situ hybridization for EBER. The feature was suggestive of fibroleiomyomatous hamartoma rather than primary leiomyoma. No recurrence has occurred in the 12 months after surgery, and the patient’s condition has remained good.

## Discussion

Apart from typical hamartomas, Spencer [4] identified three forms of hamartomatous musculofibrotic changes in the lung:

1. Single or numerous focal proliferations of the smooth muscle tissue
2. Pulmonary lymphangioleiomyomatosis
3. Tuberous sclerosis with pulmonary changes

According to Spencer, very rare focal changes occur in the walls of the pulmonary alveoli, in the pleural region, and in the walls of the small bronchi. In our case, the bronchial epithelium extended into the alveolar septa in the pulmonary parenchyma without a cartilaginous or adipose element. Spencer suggested that they mainly developed subpleurally and extended towards the hilum. In our case, they reached the subpleural space. Patients with the lesions are generally asymptomatic. Multiple lesions occur more frequently than solitary lesions, and the tumors are more prevalent in women. There are many reports on uterine leiomyomas metastasizing to the lungs [5].

The histological characteristics of what is currently referred to as fibroleiomyomatous hamartoma or benign metastasizing leiomyoma are as follows: (i) a well-circumscribed nodule composed of smooth muscle cells with numerous epithelial inclusions and (ii) no cellular atypia or mitotic figures [6, 7]. In our case, the proliferation of smooth muscle cells, which were positive for smooth muscle markers such as alpha-SMA, and the extension of bronchial epithelium into the alveolar septa were observed. Neither cellular atypia nor mitotic figures were present. There are several theories on the pathogenesis of this tumor, including (i) that it occurs as a metastasis from a benign leiomyoma or low-grade leiomyosarcoma (most often from the uterus); (ii) that it occurs due to the implantation and proliferation of benign smooth muscle tissue, which is embolized by intravenous leiomyomatosis or by mechanical means; and (iii) that they represent true fibroleiomyomatous hamartoma, originating from the lung. These tumors are not histologically distinguishable. Ito et al. reported that the terms *fibroleiomyomatous hamartoma* or *benign metastasizing leiomyoma* could include some cases of low-grade leiomyosarcomas, true benign metastasizing leiomyoma, and true fibroleiomyoma [5]. Since controversy exists about the pathogenesis of this tumor, it was thought that the current diagnostic terms of *fibroleiomyomatous hamartoma* or *benign metastasizing leiomyoma* should be reconsidered to avoid misleading diagnoses. In the present cases, the lesion was solitary and occurred in a male who had no history of smooth muscle neoplasm. Thus, *true fibroleiomyomatous hamartoma* was considered to be the most suitable the diagnostic term to describe the patient’s condition.

Four partial resections and one lobectomy are reported to have been performed in the five reported cases of pulmonary fibroleiomyomatous hamartoma. There are no reports of the postoperative recurrence of pulmonary fibroleiomyomatous hamartoma [2, 3]. We thought preoperative examinations were atypical as a malignant tumor but could not deny malignancy. Therefore, we proposed to initially perform a partial resection for this peripheral lung tumor. It was thought that lobectomy should be performed, only when frozen section had a possibility of malignancy. Surgical intervention is important, not only for achieving a cure but also as a diagnostic measure to rule out malignancy. Complete resection alone seems to be a suitable surgical treatment.

## Conclusions

We herein presented a rare resected case of a solitary pulmonary fibroleiomyomatous hamartoma and discussed its differential diagnosis and histogenesis.

## Consent

Written informed consent was obtained from the patient for publication of this case report and any accompanying images. A copy of the written consent is available for review by the Editor-in-Chief of this journal.

## Abbreviations

CT, computed tomography; SMA, smooth muscle actin; VATS, video-assisted thoracoscopic surgery
